# Performance of Damage Identification Based on Directional Wavelet Transforms and Entopic Weights Using Experimental Shearographic Testing Results

**DOI:** 10.3390/s21030714

**Published:** 2021-01-21

**Authors:** Andrzej Katunin

**Affiliations:** Department of Fundamentals of Machinery Design, Faculty of Mechanical Engineering, Silesian University of Technology, 44-100 Gliwice, Poland; andrzej.katunin@polsl.pl

**Keywords:** vibration-based damage identification, directional wavelet transforms, directional selectivity, entropy, shearography

## Abstract

The paper aims to analyze the performance of the damage identification algorithms using the directional wavelet transforms, which reveal higher sensitivity for various orientations of spatial damage together with lower susceptibility to noise. In this study, the algorithms based on the dual-tree, the double-density, and the dual-tree double-density wavelet transforms were considered and compared to the algorithm based on the discrete wavelet transform. The performed analyses are based on shearographic experimental tests of a composite plate with artificially introduced damage at various orientations. It was shown that the directional wavelet transforms are characterized by better performance in damage identification problems than the basic discrete wavelet transform. Moreover, the proposed approach based on entropic weights applicable to the resulting sets of the detail coefficients after decomposition of mode shapes can be effectively used for automatic selection and emphasizing those sets of the detail coefficients, which contain relevant diagnostic information about damage. The proposed processing method allows raw experimental results from shearography to be significantly enhanced. The developed algorithms can be successfully implemented in a shearographic testing for enhancement of a sensitivity to damage during routine inspections in various industrial sectors.

## 1. Introduction

The structural damage identification (SDI), in the light of the maintenance philosophies applied nowadays, like damage tolerance and condition-based maintenance, becomes of strategic importance, especially in the aircraft, aerospace, automotive and civil infrastructure industrial branches. Among the non-destructive testing (NDT) techniques applied for SDI in numerous problems, a class of vibration-based techniques is one of the well-established and effective approaches mainly due to their testing simplicity and cost-effectiveness as well as relatively high sensitivity to damage [1]. This class of techniques involves all testing approaches based on modal analysis, including traditional testing with vibration sensors, like accelerometers or microelectromechanical system (MEMS) sensors [2,3,4,5]. A significant enhancement of sensitivity to structural damage in such techniques was achieved by an application of non-contact measurement systems, like laser Doppler vibrometers or interferometers used in electronic speckle pattern interferometry or shearography, which allowed the precision of measured quantities to be increased by up to several orders with respect to traditional contact sensors [6,7,8,9,10]. The details on the main principles of operation of shearography as well as the areas of potential applications can be found in review papers [11,12,13]. However, despite the increased measurement accuracy which can be obtained using the mentioned measurement devices, the problem of identification a small structural damage remained open, which forced numerous research teams to develop post-processing algorithms for enhancing damage detectability with respect to raw measurement results. These algorithms are applied to the mode shapes of vibration due to the proven sensitivity of this modal parameter to structural damage [1,5].

One of the widely employed and most effective post-processing algorithms used in SDI problems is a class of algorithms based on wavelet transforms (WTs) of the mode shapes of vibration. This class of algorithms owes its effectiveness to the high sensitivity to signal disturbances, which, in the case of SDI problems, is related to a local stiffness decrease due to the presence of damage. The great advantage of such an approach is a baseline-free analysis, i.e., the damage identification is performed on a damaged structure only, without a necessity of comparing the obtained results to the results of an intact structure. This is possible due to the high sensitivity of WTs to the mentioned local stiffness decreases caused by a damage. Numerous approaches based on continuous wavelet transform (CWT) and discrete wavelet transform (DWT) were developed to date (see, e.g., [6,14,15,16,17,18,19,20,21,22,23]). However, using the mentioned WTs in the 2D SDI problems, namely, in identification of damage in plate-like structures, introduces some limitations in the directional selectivity of the identified damage. In other words, the mentioned WTs have predefined directional coefficients: horizontal, vertical, and diagonal, and when the orientation of a damage differs with respect to these predefined directions, its detectability decreases significantly, which was discussed, e.g., in [24].

To overcome the problem of decreased sensitivity for a damage with orientation different to the predefined directions of wavelet coefficients, only several investigations were performed to date. The problem was studied by the research groups of Ostachowicz and Cao [24,25], where the authors employed the complex WT with directional Gaussian wavelets to increase the directional sensitivity to structural damage in the investigated homogeneous and heterogeneous plates. Another approach with using the curvelet transform revealing sensitivity to various orientations of damage was proposed by the research group of Bagheri [26,27]. Several studies on the enhancement of directional selectivity to specifically oriented damage in 2D structures was performed by the author’s team [28,29,30], where the complex fractional WT and quaternion WT were applied for post-processing mode shapes of investigated structures. Nevertheless, systematic studies on the selection and analysis of WTs suitable for effective damage identification considering its orientation were not performed to date.

The class of directional WTs was developed by Kingsbury and Selesnick [31,32,33,34,35,36], which contains three general types of WTs: the dual-tree WT (DTWT), double-density WT (DDWT), dual-tree double-density WT (DTDDWT), achieved by introducing a complexification of the classical DWT. These transforms, in such a way, obtained numerous advantageous properties with respect to DWT, namely, better directional sensitivity and substantial noise reduction [36]. Following this, they found numerous applications, primarily in image processing problems in various studies [37,38,39,40,41,42,43], but also in machinery condition monitoring [44,45,46,47,48].

The decomposition using the above-mentioned directional WTs results in numerous sets of wavelet coefficients—in the most complex case, when the real 2D DTDDWT is applied to a 2D signal, one obtains 16 sets of wavelet coefficients [34]. Assuming that the location, shape, and orientation of a damage is not known a priori, it is essential to select only the relevant sets of wavelet coefficients to identify damage. This becomes an important processing step, when multiple mode shapes are considered in the analysis, which characterize by various sensitivity to damage. The approach used, e.g., in [20,49], when all sets of wavelet coefficients are summed up, becomes to be not effective, since the sets of coefficients, which do not contain useful diagnostic information, decrease the detectability of damage due to adding noise and masking the information from coefficients containing information about damage. This implies the necessity of a selection of relevant sets of wavelet coefficients using a quantitative approach. One of such approaches is an evaluation of relevancy by means of entropic measures originated from the wavelet packet transform (WPT) and the related best-tree wavelet packet analysis introduced by Coifman and Wickerhauser [50], which is based on a selection of the sets of coefficients after WPT decomposition based on its energy or entropy. Such measures found an application in SDI problems in direct damage detection and identification, i.e., the mentioned measures were used for the construction of damage indices applied for identification of a damage. This approach was adapted to SDI problems in numerous research studies. Ren and Sun [51] used wavelet entropy (WE) for the identification of damage in beams, determining entropy from the measured time histories at the measurement points. A similar approach using WPT was presented in [52,53]. The improvement of this approach was proposed in [54], where the authors defined two entropy measures using probability analysis for the identification and quantification of cracks in beams. Another approach was proposed in [55,56], where the authors determined entropy of the frequency response spectra of the tested beam- and plate-like structures. The authors of [57] developed an entropy-based index, which was determined from the difference in modal curvatures of intact and damaged beams.

The aim of this paper is to evaluate the performance of the mentioned directional wavelet transforms accompanied with the new technique of an automated selection of sets of wavelet coefficients after decomposition of mode shapes based on entropic weights. In particular, the sensitivity to damage sites of various spatial orientations is examined. The novel algorithm with enhanced directional selectivity and automatized selectivity of relevant sets of wavelet coefficients reveals significant enhancement of detectability of damage, which was presented in comparative studies. The analysis was performed on experimental data obtained using shearography NDT technique of a composite plate with artificially introduced damage.

## 2. Theoretical Background and Proposed Damage Identification Algorithm

This section aims to present an outline of the theoretical background for the considered directional WTs in order to show their main properties and the way of definition of detail coefficients obtained from the decomposition of 2D signals. Moreover, according to the announced approach of using WE for automatic selection of relevant diagnostic information about structural damage from the detail coefficients, the theoretical background also addresses to the concept of WE. Finally, based on the presented overview of both concepts, the description of the proposed damage identification algorithm was provided at the end of this section.

### 2.1. Directional Wavelet Transforms

Considering a 2D signal s(x,y), the single-level decomposition following the classical DWT algorithm has the following form:

(1) $$s\left(x,y\right)=\overset{\infty }{\underset{k_{1}=-\infty }{\sum }}\overset{\infty }{\underset{k_{2}=-\infty }{\sum }}a\left(k_{1},k_{2}\right)\Phi \left(x,y\right)+\overset{3}{\underset{i=1}{\sum }}\overset{\infty }{\underset{k_{1}=-\infty }{\sum }}\overset{\infty }{\underset{k_{2}=-\infty }{\sum }}d^{\left(i\right)}\left(k_{1},k_{2}\right)\Psi^{\left(i\right)}\left(x,y\right),$$

where a(·) and $d^{\left(i\right)}\left(\cdot \right)$ are the sets of the approximation and detail wavelet coefficients, and the 2D scaling Φ(x,y) and wavelet $\Psi^{\left(i\right)}\left(x,y\right)$ functions are defined by permutation of tensor products of 1D scaling φ(x) and wavelet ψ(x) functions as follows:

(2) Φ(x,y)=φ(x)φ(y),

(3) $$\Psi^{\left(1\right)}\left(x,y\right)=\varphi \left(x\right)\psi \left(y\right),\;\Psi^{\left(2\right)}\left(x,y\right)=\psi \left(x\right)\varphi \left(y\right),\;\Psi^{\left(3\right)}\left(x,y\right)=\psi \left(x\right)\psi \left(y\right),$$

where (i) denotes the orientation of a wavelet function: horizontal (0°), vertical (90°), and diagonal (±45°), respectively.

In the case of DTWT, the particular 1D wavelets has a complex (approximately analytic) form, i.e., $\tilde{\psi }\left(x\right)=\psi_{h}\left(x\right)+j\psi_{g}\left(x\right)$, where h(x) and g(x) represent low-pass and high-pass filters, respectively, and $j=\sqrt{-1}$. Following this, Equation (3) takes the form [36]:

(4) $$\Psi^{\left(1,1\right)}\left(x,y\right)=\varphi_{h}\left(x\right)\psi_{h}\left(y\right)\;,\;\Psi^{\left(2,1\right)}\left(x,y\right)=\varphi_{g}\left(x\right)\psi_{g}\left(y\right),\;\Psi^{\left(1,2\right)}\left(x,y\right)=\psi_{h}\left(x\right)\varphi_{h}\left(y\right)\;,\;\Psi^{\left(2,2\right)}\left(x,y\right)=\psi_{g}\left(x\right)\varphi_{g}\left(y\right),\;\Psi^{\left(1,3\right)}\left(x,y\right)=\psi_{h}\left(x\right)\psi_{h}\left(y\right)\;,\;\Psi^{\left(2,3\right)}\left(x,y\right)=\psi_{g}\left(x\right)\psi_{g}\left(y\right),$$

where the indices $\left(i_{1},i_{2}\right)$ denote the decomposition tree: $i_{1}=1$ for real, and $i_{2}=2$ for imaginary ones, and $i_{2}=1,2,3$ for particular orientations, namely ±15° for $\Psi^{\left(1,1\right)}$ and $\Psi^{\left(2,1\right)}$, ±45° for $\Psi^{\left(1,2\right)}$ and $\Psi^{\left(2,2\right)}$, and ±75° for $\Psi^{\left(1,3\right)}$ and $\Psi^{\left(2,3\right)}$, respectively, which allows strongly oriented wavelets to be obtained in 6 directions.

Another extension of DWT, the DDWT, was proposed by Selesnick in [33], introducing the set of three functions, instead of two in the classic DWT: scaling φ(x) and wavelet ψ(x) functions. The concept of DDWT is based on the offset of the first wavelet function to one another by one half as follows:

(5) $$\psi_{h,1}\left(x\right)\approx \psi_{g,2}\left(x-0.5\right),\;\psi_{g,1}\left(x\right)\approx \psi_{h,2}\left(x-0.5\right),$$

thus, in this case, we operate with φ(x), and $\psi_{1}\left(x\right)$ and $\psi_{2}\left(x\right)$, which allows decreasing the shift sensitivity of DWT and susceptibility to noise. According to the above-presented formulation, in the case of 2D DDWT, one obtains 8 wavelet functions due to permutation of mentioned 1D scaling and wavelet functions:

(6) $$\Psi^{\left(1\right)}\left(x,y\right)=\varphi \left(x\right)\psi_{1}\left(y\right)\;,\;\Psi^{\left(2\right)}\left(x,y\right)=\varphi \left(x\right)\psi_{2}\left(y\right),\;\Psi^{\left(3\right)}\left(x,y\right)=\psi_{1}\left(x\right)\varphi \left(y\right)\;,\;\Psi^{\left(4\right)}\left(x,y\right)=\psi_{2}\left(x\right)\varphi \left(y\right),\;\Psi^{\left(5\right)}\left(x,y\right)=\psi_{1}\left(x\right)\psi_{1}\left(y\right)\;,\;\Psi^{\left(6\right)}\left(x,y\right)=\psi_{1}\left(x\right)\psi_{2}\left(y\right),\;\Psi^{\left(7\right)}\left(x,y\right)=\psi_{2}\left(x\right)\psi_{1}\left(y\right)\;,\;\Psi^{\left(8\right)}\left(x,y\right)=\psi_{2}\left(x\right)\psi_{2}\left(y\right).$$

The last transform discussed here, the DTDDWT, combines DTWT and DDWT proposed in [34,35], which combines the properties of directional selectivity of DTWT as well as near shift-invariance and reduced susceptibility to noise. Applying Equation (5) to Equation (4), one obtains 16 wavelets in each decomposition tree, thus 32 wavelets in total.

The details on the construction of filterbanks for these three WTs can be found in the original works of Selesnick and Kingsbury [31,32,33,34,35,36].

### 2.2. Wavelet Entropy

The concept of WE was adapted from the original study of thermodynamic entropy established by Shannon [58] and aimed to measure the thermodynamic disorder in signals. In wavelet analysis, this concept is used as an information disorder measure of any statistical distribution, which makes it possible to evaluate quantitatively information in the sets of the wavelet coefficients after WT-based signal decomposition, as it was mentioned in Section 1. According to the Shannon’s formulation [58], entropy S can be represented as follows:

(7) $$S=-\overset{N}{\underset{i=1}{\sum }}p_{j}\ln\left(p_{j}\right),$$

where $p_{i}$ is the probability of an output j, whereas N is a total number of probable outputs. In the context of WE, p represents the wavelet energy ratio of a given set of wavelet coefficients $d^{\left(i\right)}$ obtained after decomposition to the total energy of all sets of wavelet coefficients given by:

(8) $$p=\frac{{\left|d^{\left(i\right)}\right|}^{2}}{\sum_{i=1}^{I}{\left|d^{\left(i\right)}\right|}^{2}},\;\;i=1,\ldots ,I.$$

Using Equation (8), it is possible to quantify the complexity of particular sets of wavelet coefficients in a quantitative way, such that the higher S, the more complex is the given set of coefficients.

The classical formulation of Shannon was extended in different manners according to the necessities of evaluation of information complexity. One of such extensions is WE based on the Stein’s Unbiased Risk Estimate (SURE), adapted by Donoho and Johnstone [59,60] and based on the original work of Stein [61]:

(9) $$S\left(\varepsilon \right)=P-\#\left\{j:\left|s_{j}\right|\leq \varepsilon \right\}+\overset{P}{\underset{j=1}{\sum }}\min\left(s_{j}^{2},\varepsilon^{2}\right),$$

where $s_{j}$ is the jth sample of the signal, ε>0 is the threshold value, and P is the length of the signal. It is worth to note that the SURE WE provides better distinguishability in the determined entropy of particular sets of wavelet coefficients than the classical Shannon’s entropy formulation.

### 2.3. Damage Identification Algorithm

The developed SDI algorithm combines the oriented WTs with SURE WE used here to determine the weights during summation of particular sets of the wavelet coefficients. This approach allows automation of selection the relevant information about structural damage consisted in the wavelet coefficients after decomposition of mode shapes.

**Figure 1:**
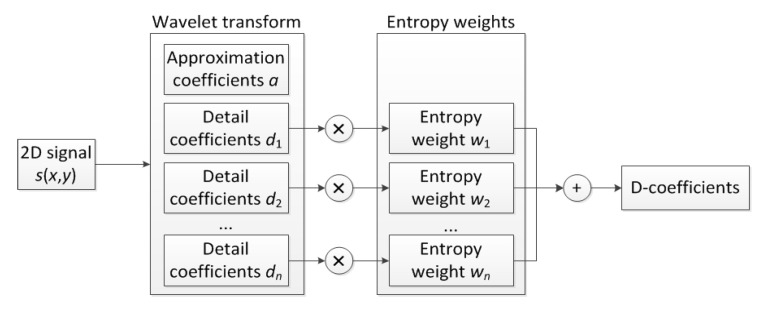
Flowchart of the processing algorithm.

Considering a set of 2D mode shapes $\left\{m_{1}\left(x,y\right),m_{2}\left(x,y\right),\ldots ,m_{n}\left(x,y\right)\right\}$ and the resulting sets of detail coefficients obtained from their decomposition using the oriented WTs described in Section 2.1 $d_{t}^{\left(i\right)}$, t=1,…,n, and taking into consideration SURE WE weights $w_{t}\left(S\left(\varepsilon \right)\right)$, one can define the summation formula as follows:

(10) $$D=\overset{n}{\underset{t=1}{\sum }}\overset{I}{\underset{i=1}{\sum }}\frac{{\left(d_{t}^{\left(i\right)}\right)}^{\alpha }}{w_{t}{\left(S\left(\varepsilon \right)\right)}^{\beta }},$$

where α and β are the scaling parameters used for enhancement of distinguishability of identified damage signatures and increasing the distinguishability of entropy weights for particular sets of detail coefficients, respectively. The entropic weights in the denominator in (10) aimed to enhance those sets of detail coefficients which have lower entropy. This is due to the fact that higher entropy is typical for detail coefficients with low-valued or no damage signatures, thus, they are dominated by noise. The flowchart for the determination of the D-coefficients according to Equation (10) is presented in Figure 1.

## 3. Materials, Testing Procedure and Data Pre-Processing

**Figure 2:**
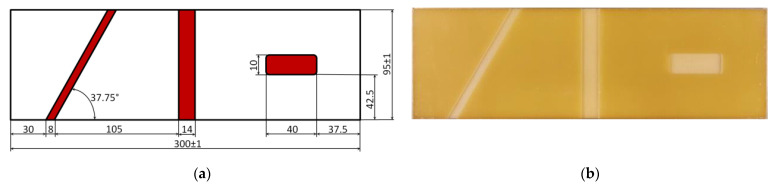
The geometry of the tested specimen: (**a**) scheme, (**b**) view.

The tests were performed on the glass fiber-reinforced composite plate with the symbol EP GC 201 manufactured and supplied by Izo-Erg S.A. (Gliwice, Poland) with the following dimensions: the length of 300 ± 1 mm, the width of 95 ± 1 mm, and the thickness of 2.38 mm. The details on the constituents, manufacturing process, and the mechanical properties of the plate can be found in [62]. Furthermore, the artificial damage was introduced into the plate using the milling machine according to the scheme presented in Figure 2a. The view of the damaged plate is presented in Figure 2b. The thickness reduction in the milled regions was of 1.19 mm (50% of a total thickness). The introduced damage sites have the following characteristics: the middle damage (the second damage from the left) is of a through-the-width type, the left-side damage (the first damage from the left) is of an angular through-the-width type with an angle different from 45°, and the right-side damage (the third damage from the left) was of internal type. Such a combination of damage sites allowed to test the performance of the investigated oriented WTs, which is discussed in the further part of this paper.

**Figure 3:**
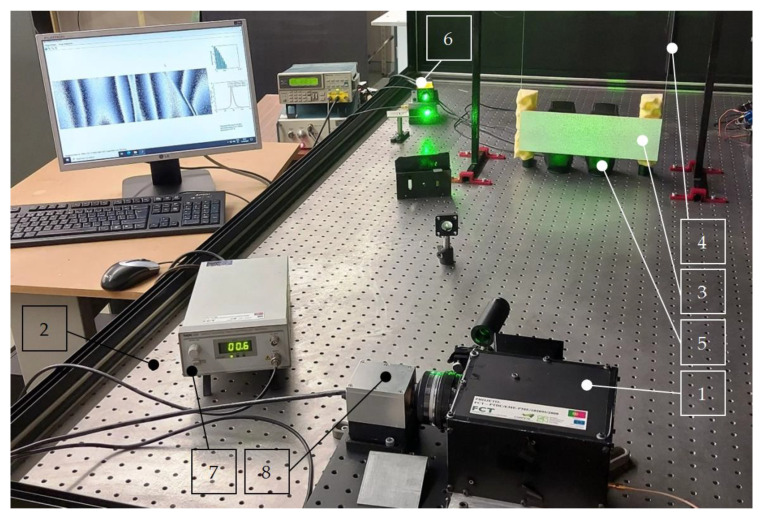
The experimental setup of shearographic measurements.

**Figure 4:**
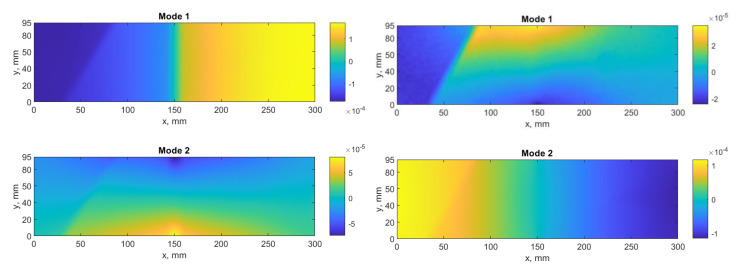

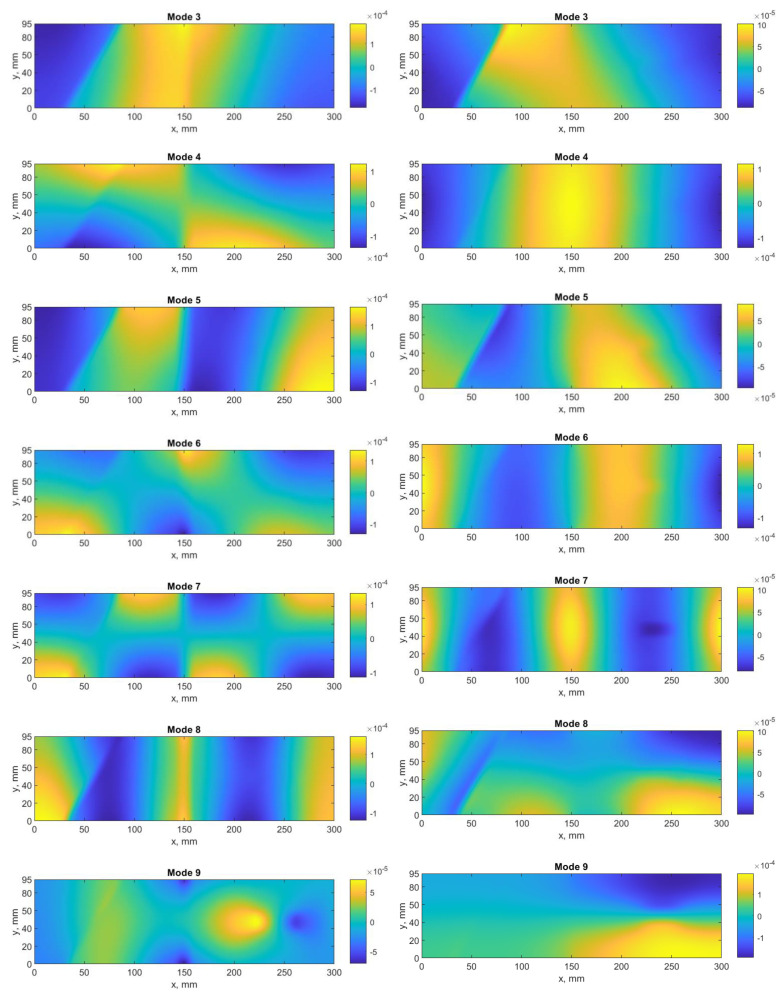

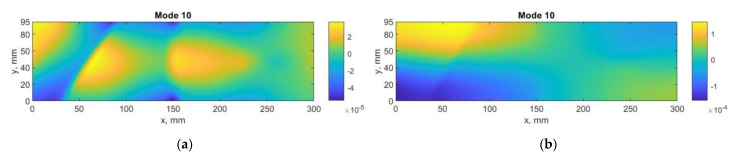
The considered mode shapes for: (**a**) *x*-direction, (**b**) *y*-direction measurements.

The shearographic testing procedure was performed in a similar way as it was described in [63] using a custom-made shearographic system *1* mounted on an optical table *2* to isolate the measurement system from external vibrations. The tested specimen *3* was hanged on two very flexible wires *4* from both sides to simulate free-free boundary conditions (see Figure 3) and excited acoustically using loudspeakers *5* with acoustic signals corresponding to the previously identified natural frequencies of the tested specimen (for more details on the procedure of identification of the natural frequencies see [63]). The tests were performed in the frequency range of 0–1.6 kHz, and 10 modes were considered for further investigations. The number of modes taken into consideration in this study was determined in an empirical way to obtain modal response of the tested plate for all three introduced damage sites. Since the structural response for the right-side damage became observable from the 9th mode shape (see Figure 4), the number of the considered mode shapes is limited to 10. The identified natural frequencies are presented in Table 1.

The measurements were performed using the 2 W (at 532 nm) continuous wave laser Coherent^®^ Verdi V Series (Santa Clara, CA, USA) *6*. For achieving the uniform reflection of a laser light, the surface of the specimen was covered with a thin layer of white powder on the back side with respect to the surface with introduced damage. The custom-made shearographic system *1* used for measuring modal rotations is based on the optical Michelson interferometer connected to piezoelectric actuator responsible for translation of a mirror in the interferometer and controlled by the controller *7* and a high-resolution CCD camera *8*. The modal rotations of the tested specimen were measured at their highest amplitudes by using the temporal phase modulation technique described in detail in [63]. The measurements were performed in *x* and *y* directions with the shearing amount of 5 mm.

The acquired phase maps from the performed tests were wrapped within [−π,π] and biased by high-frequency noise. To obtain continuous phase maps for modal rotations, the filtering procedure was performed using the sine/cosine and 3D filters with windows of 5 × 5 and 25 × 25 samples, respectively, and then the unwrapping procedure was applied using the Goldstein algorithm (see [63] for more details). The spatial resolution for the modal rotations obtained for *x*- and *y*-direction measurements was 1251 × 3964 and 1177 × 4028, respectively. The resulting modal rotation fields considered in further analyses are presented in Figure 4.

From the above-presented modal rotation fields for the considered mode shapes, one can distinguish bare signatures of the introduced damage sites. In particular, the first and second damage sites can be detected from disturbances of modal rotation fields in the most cases, while the third damage site is detectable only from the several high-order modal rotation fields. However, the identification of these damage sites is not possible at the above-described step of pre-processing. Following this, the algorithms based on the considered directional wavelet transforms are analyzed in the next section.

## 4. Analysis of Performance of Damage Identification Algorithms

In this study, four algorithms based on wavelet transforms were taken into consideration: the algorithms based on the 2D DWT, as the initial algorithm, and three algorithms based on the directional WTs, namely, 2D DTWT, DDWT, and DTDDWT. Due to the requirements of these WTs to the dimensions of 2D signals, which should be the powers of 2, the considered mode shapes presented in Figure 4 were extended to the dimensions of 2048 × 4096 samples by adding zeros in both directions, and after performing decomposition, the resulting sets of detail coefficients were trimmed to the dimensions of 626 × 1982 for *x*-direction and 589 × 2014 for *y*-direction measurements, respectively. The reduction of the dimensions of the resulting sets of the detail coefficients is the effect of downsampling procedure typical for all considered WTs.

### 4.1. Algorithm Based on DWT

**Figure 5:**
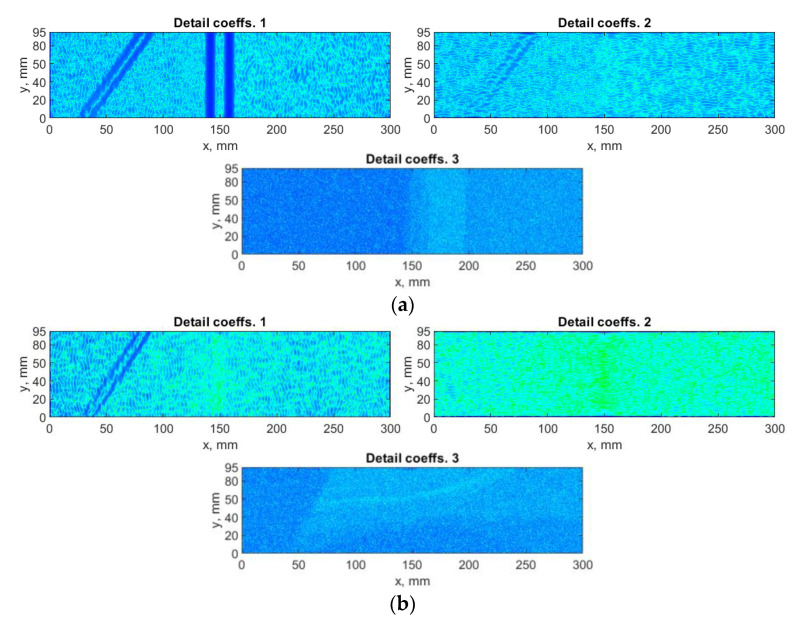
The exemplary results of decomposition using discrete wavelet transform (DWT)-based algorithm for the first mode shape: (**a**) *x*-direction, (**b**) *y*-direction measurements.

**Figure 6:**
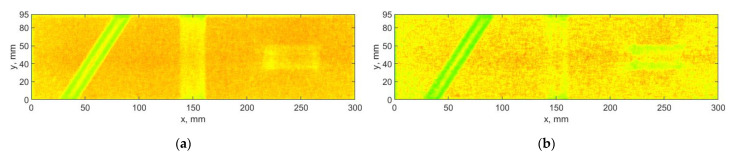
The results of processing the mode shapes for the DWT-based algorithm: (**a**) without using, and (**b**) with using of the SURE WE weights.

The algorithm based on DWT was applied to the considered mode shapes with taking into consideration the summation approach for the resulting detail coefficients obtained for the particular mode shapes described in Section 2.3. Each mode shape is decomposed to three sets of detail coefficients, which correspond with three orientations defined by Equation (3). The Farras nearly symmetric filters together with the Q-shift Kingsbury filters were applied in the decomposition process. The exemplary results for a decomposition of the selected mode shape and the results of summation with and without using the SURE WE weights are presented in Figure 5 and Figure 6, respectively. In the following and all the next analyzes the threshold value ε, and the parameters α and β in Equation (10) were assumed as 5, 0.01, and 50, respectively. These values were determined in an empirical way. Due to the difference of the spatial dimensions for various measurement directions (see Section 3 for more details), the dimensions of the resulting sets were adjusted to the lower dimensions in each direction during summation of the sets of the detail coefficients for various measurement directions.

From the results of decomposition using the DWT-based algorithm (see example in Figure 5), one can observe that mainly the horizontal and vertical detail coefficients contain a diagnostic information about damage, while the diagonal detail coefficients contain mainly noise, which is added to the final set D, when SURE WE weights are not considered (see Figure 6a). To overcome this deficiency, the SURE WE weights were applied for the determination of the final set D, which is presented in Figure 6b. It can be observed that the proposed approach which considers SURE WE weights allowed enhancing the detectability of the introduced damage, especially the third damage, which was barely detectable for the case when the weights were not applied (cf. Figure 6a,b).

### 4.2. Algorithm Based on DTWT

**Figure 7:**
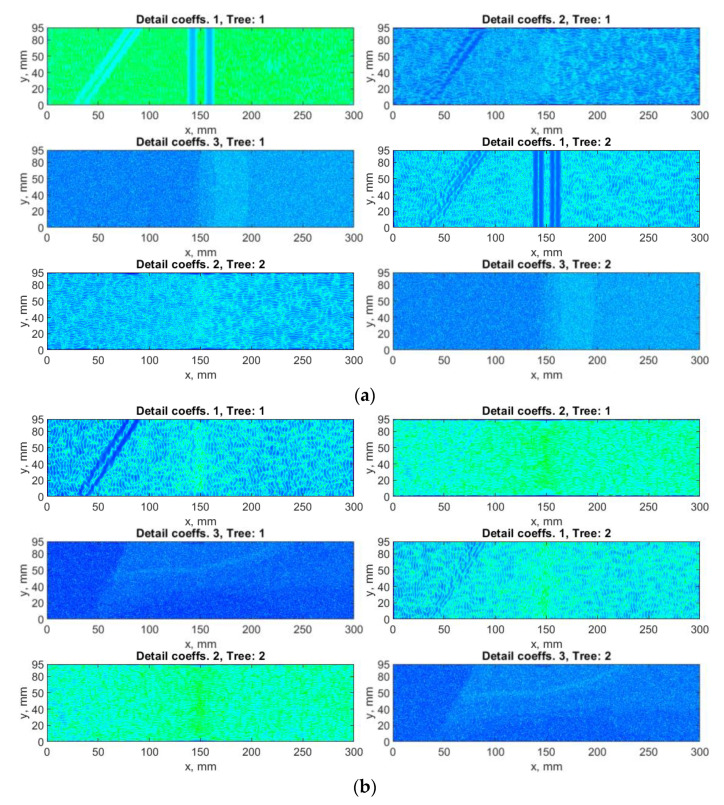
The exemplary results of decomposition using dual-tree WT (DTWT)-based algorithm for the first mode shape: (**a**) *x*-direction, (**b**) *y*-direction measurements.

**Figure 8:**
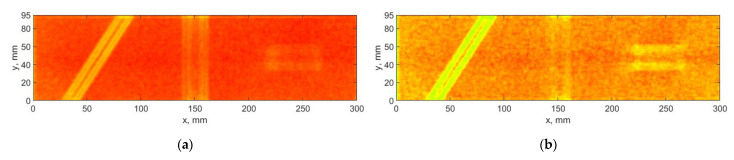
The results of processing the mode shapes for the DTWT-based algorithm: (**a**) without using, and (**b**) with using of the SURE WE weights.

The first directional WT-based algorithm considered in this study was based on real DTWT. The decomposition of the considered mode shapes was performed using the Farras filters and the 6-tap Kingsbury Q-shift filters selected in an empirical way, which resulting in six sets of the detail coefficients according to Equation (4). The results of decomposition of the selected mode shape are presented in Figure 7, while the D sets determined with and without considering SURE WE weights is presented in Figure 8.

The application of the DTWT-based algorithm resulting in some sets of the detail coefficients insensitive to the introduced damage, which confirms the necessity of considering the weights for the obtained sets of detail coefficients. A significant improvement is observable for the result presented in Figure 8b, especially for the third damage, which is almost undetectable when the SURE WE weights were not applied (see Figure 8a). Moreover, one can observe significant reduction of noise and better detectability of the boundaries of the damage sites, especially in the case of second and third damage (cf. Figure 6 and Figure 8).

### 4.3. Algorithm Based on DDWT

**Figure 9:**
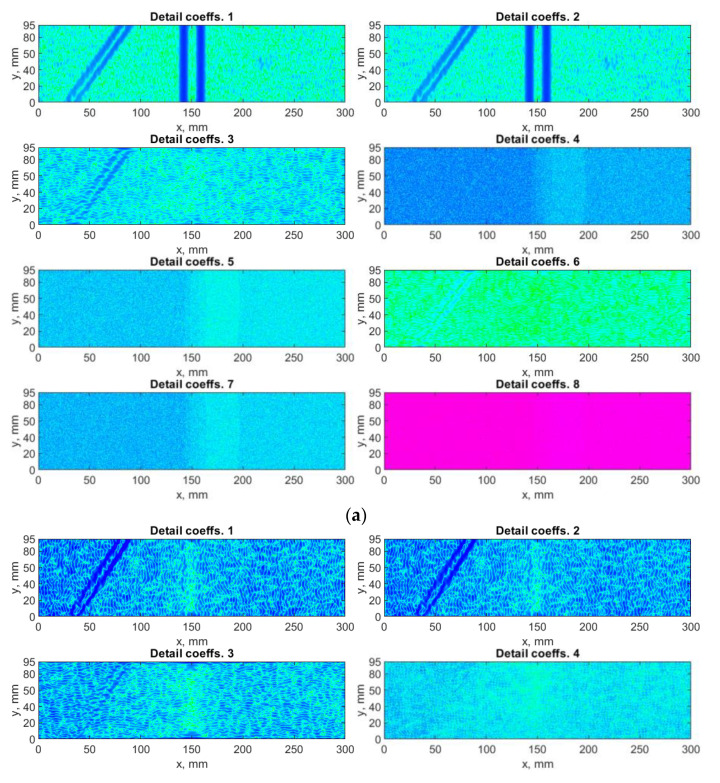

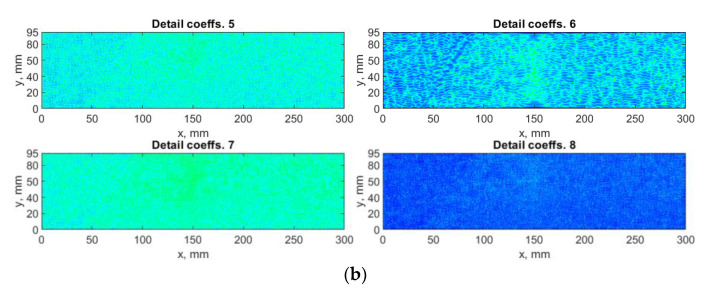
The exemplary results of decomposition using double-density WT (DDWT)-based algorithm for the first mode shape: (**a**) *x*-direction, (**b**) *y*-direction measurements.

**Figure 10:**
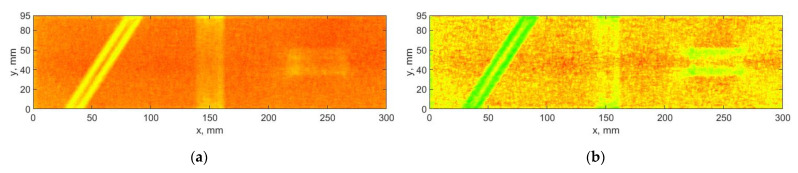
The results of processing the mode shapes for the DDWT-based algorithm: (**a**) without using, and (**b**) with using of the SURE WE weights.

Next, the DDWT-based algorithm was applied for the decomposition of the considered mode shapes using the 6-tap Kingsbury Q-shift filters selected in an empirical way, which resulting in eight sets of the detail coefficients according to Equation (6). The results of decomposition of the selected mode shape using this algorithm are presented in Figure 9. The D sets obtained from the summation according Equation (10) with and without taking into consideration the SURE WE weights are shown in Figure 10.

The above-presented results show that the influence of the sets of the detail coefficients containing noise in the case of the DDWT-based algorithm is significant, which resulted in masking the boundaries of the damage signatures in the D sets (see Figure 10a). Although, the damage signatures for the second damage are clearly detectable in several first sets of coefficients (see Figure 9), the resulting D sets are characterized by lower detectability of damage boundaries. However, the improvement of the D sets considering the SURE WE weights is noticeable in this case (cf. Figure 10a,b), especially for the second and third damage, where the boundaries of the damage signatures become to be distinguishable. Moreover, the obtained results presented in Figure 10b reveal better distinguishability of damage compared to the previous results obtained with the DWT- and DTWT-based algorithms (cf. Figure 6b, Figure 8b and Figure 10b).

### 4.4. Algorithm Based on DTDDWT

**Figure 11:**
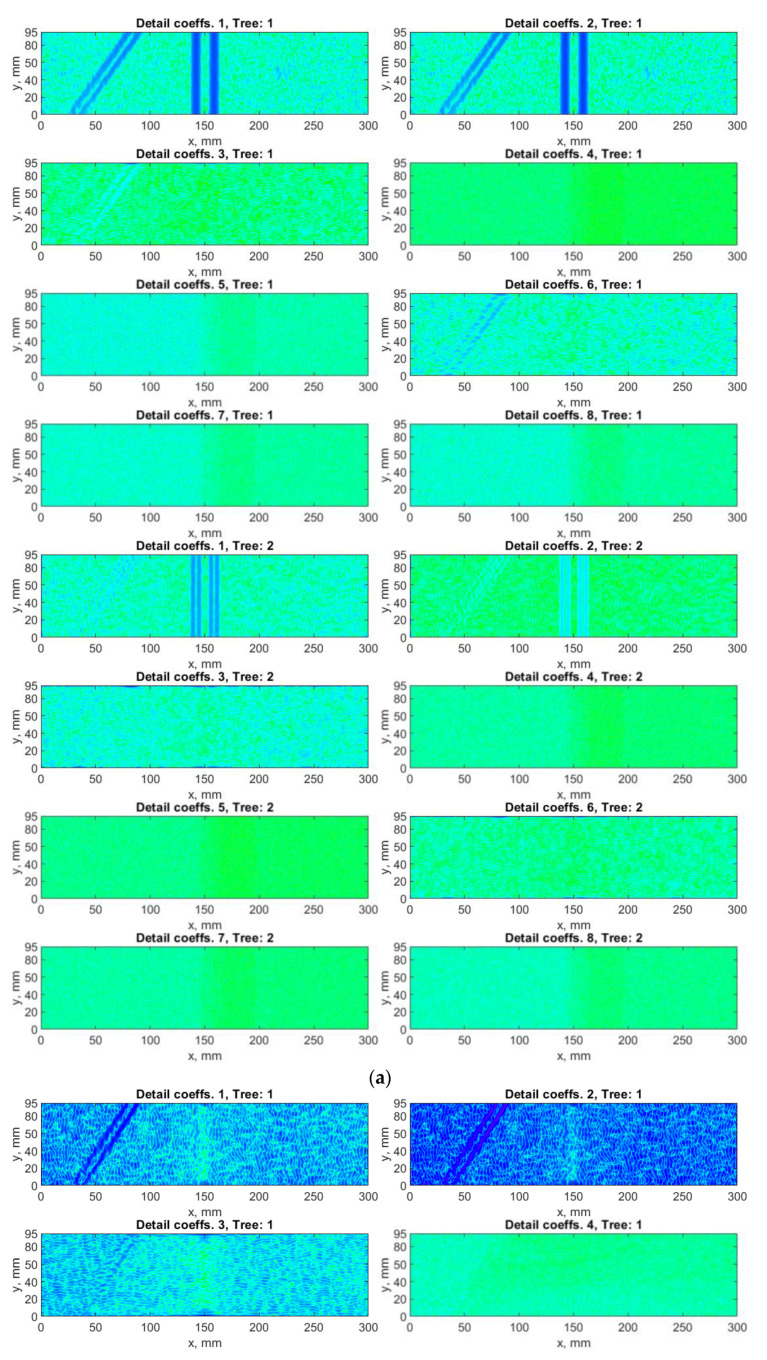

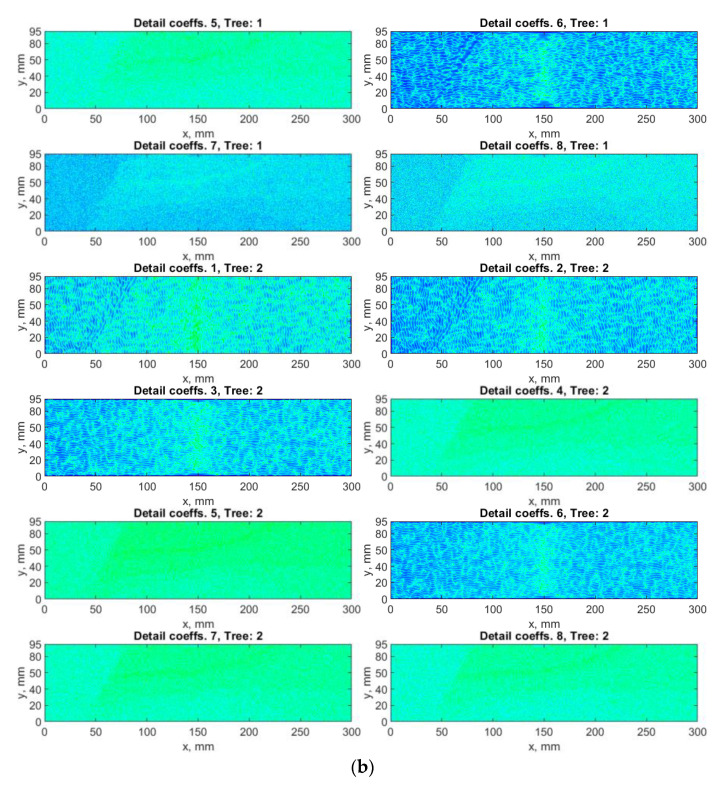
The exemplary results of decomposition using dual-tree double-density WT (DTDDWT)-based algorithm for the first mode shape: (**a**) *x*-direction, (**b**) *y*-direction measurements.

**Figure 12:**
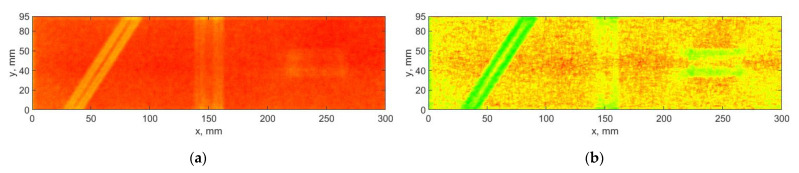
The results of processing the mode shapes for the DTDDWT-based algorithm: (**a**) without using, and (**b**) with using of the SURE WE weights.

The last considered directional WT-based algorithm was implemented using the real DTDDWT with the empirically selected 10-tap Kingsbury Q-shift filters, which due to the combination of DTWT and DDWT, whose wavelet functions are represented by Equations (4) and (6), respectively, resulted in sixteen sets of the detail coefficients. The resulting sets of the detail coefficients after the decomposition are presented in Figure 11, while the results of summation of the obtained detail coefficients for the considered mode shapes are shown in Figure 12.

The obtained results from the decomposition procedure (Figure 11) show, in general, that the number of the sets of the detail coefficients sensitive to damage is proportional to the results of the decomposition using the DDWT-based algorithm. The improvement of damage detectability when the SURE WE weights were considered is noticeable (cf. Figure 12a,b), which can be explained by the increasing number of the sets of the detail coefficients, which mask the damage signatures, when the weights are not taken into consideration. However, comparing the results presented in Figure 12b with those obtained with the DDWT-based algorithm (Figure 10b), it can be stated that no visible improvement of damage detectability using the DTDDWT-based algorithm is observed.

### 4.5. Performance Evaluation and Discussion

One of the advantages of the considered algorithms based on the directional WTs with respect to DWT is their robustness to noise, which is observable from a comparison of the resulting D sets without considering the SURE WE weights for DWT-based algorithms (see Figure 6a) and the algorithms based on the directional WTs (see Figure 8a, Figure 10a and Figure 12a). This also finds a confirmation in the results obtained with taking into consideration of the SURE WE weights, where the intensity of the damage signatures is higher with respect to those obtained with the DWT-based algorithm.

**Figure 13:**
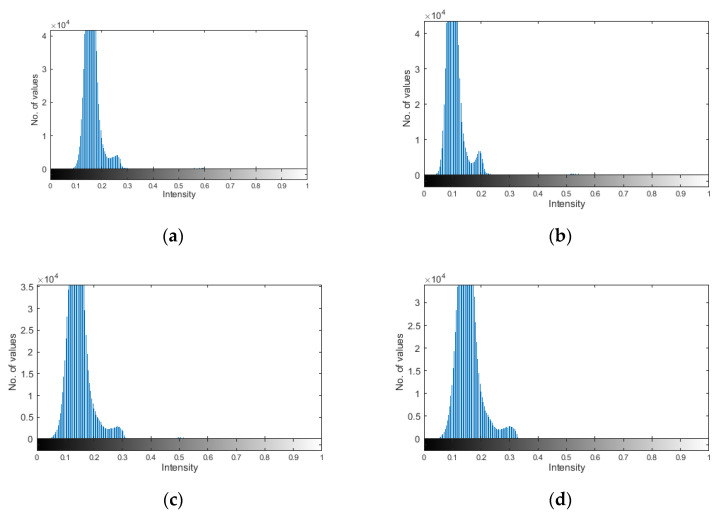
The histograms of the normalized D sets for: (**a**) DWT-, (**b**) DTWT-, (**c**) DDWT-, and (**d**) DTDDWT-based algorithms.

In order to confirm this statement quantitatively, the following procedure was implemented. The D sets with a consideration of the SURE WE weights were normalized and the two-level Otsu’s thresholding was applied in order to separate the damage signatures from the background noise. The Otsu’s thresholding is the authomatic thresholding method, which transforms an image to the selected number of classes. In the most simplest case, Otsu’s thresholding method returns one level and a binary image classified based on this level. The selection of two levels is resulted from the distribution of the values in the D sets, which can be observed in the histograms presented in Figure 13. The resulting thresholds are presented in Table 2. Next, based on the determined thresholds, the values of the D sets were assigned to two classes representing the damage signatures and the background noise. Finally, the sum of all values from the class representing the damage signatures was divided by the sum of all values from the class representing the background noise. The obtained metric allowed evaluating the intensity of the damage signatures with respect to the background noise. The results of calculations are presented in Table 2. As it can be observed, the obtained values of the intensity metric clearly indicate the increased intensity for all the considered algorithms based on the directional WTs, which confirms better damage detectability of these algorithms.

In order to evaluate a performance of the considered WT-based algorithms in a perceptual way, another metric was used, namely, the Natural Image Quality Evaluator (NIQE) proposed by Mittal et al. [64] and adapted in this study. This quality evaluator falls into the class of the no-reference image quality assessment metrics used in numerous applications of image processing and quality evaluation (see, e.g., [65,66,67,68]). In contrast to the Blind/Referenceless Image Spatial Quality Evaluator (BRISQUE) from the same class of metrics, NIQE is opinion-unaware metric, i.e., it is not based on subjective quality scores. NIQE is a perceptual metric, which is based on a construction of features from a corpus of natural images, and then fitting them to a multivariate Gaussian (MVG) model, which can be represented as follows [64]:

(11) $$\mathrm{NIQE}=\sqrt{{\left(v_{1}-v_{2}\right)}^{T}\left(\frac{\Sigma_{1}+\Sigma_{2}}{2}\right)\left(v_{1}-v_{2}\right)},$$

where $v_{1}$ and $v_{2}$ are the mean vectors, and $\Sigma_{1}$ and $\Sigma_{2}$ are the covariance matrices of the natural MVG model and the model of a considered image, T means the transposition operation on vectors. The lower the score of NIQE, the better perceptual quality of an analyzed image.

The above-presented properties of NIQE make it useful for the qualitative evaluation of the resulting D sets obtained with using various WT-based algorithms considering the summation approach Equation (10). The results of calculation of NIQE for these cases are presented in Table 3.

The determined NIQE scores confirmed the previous results represented by the intensity metric. It can be stated that DDWT- and DTDDWT-based algorithms reveal the best damage detectability from the considered WT-based algorithms. However, the confirmed better damage detectability of the two latter algorithms is at the cost of increasing computational complexity with respect to DWT-based one.

## 5. Conclusions

In this study, the performance of the directional WTs in SDI problems was evaluated based on the experimental shearographic testing results of the composite plate with the artificially introduced damage sites. The main advantages of the considered directional WTs is their increased sensitivity to damage and robustness to noise, which is resulting in an improvement of damage identification in structures. This improvement was confirmed using the intensity and NIQE metrics, which allowed proving it quantitatively. It was shown that both metrics used for the evaluation of the damage identification sensitivity revealed a strong improvement with respect to the DWT-based algorithm, especially the DDWT and DTDDWT-based algorithms. In the case of the intensity metric the improvement was of ca. 30% (see Table 2), while in the case of the perceptual metric the improvement was of ca. 20% (see Table 3). Moreover, the proposed summation approach based on the SURE WE weights allowed considering only relevant diagnostic information from the detail coefficients of the particular mode shapes, which makes it possible to construct a single set containing all relevant damage signatures representing structural damage. The improvement of the resulting sets representing structural damage using the SURE WE weights was confirmed in numerous studies by gaining the detectability of damage. The obtained results indicated that the proposed method is a strong and viable approach in SDI problems and due to the advantageous properties of the directional WTs which can be successfully applied to SDI problems with various damage locations and orientations in practice, where shearographic testing can be applied.

## Figures and Tables

**Table 1:** Natural frequencies of the tested specimen.

Number	1	2	3	4	5	6	7	8	9	10
*f*, Hz	73.8	137.1	252.1	303.1	441.4	506.0	776.5	850.4	951.1	1018.2

**Table 2:** The determined Otsu’s threshold values and the intensity metric values.

Algorithm	DWT	DTWT	DDWT	DTDDWT
Threshold 1, –	0.1882	0.1333	0.1725	0.1882
Threshold 2, –	0.4000	0.3804	0.3980	0.4078
Intensity metric, –	0.2074	0.2536	0.3169	0.3000

**Table 3:** The NIQE scores for the D sets obtained with the considered WT-based algorithms.

Algorithm	DWT	DTWT	DDWT	DTDDWT
NIQE, –	5.8497	6.0903	4.6318	4.6998

## Data Availability

The raw/processed data required to reproduce these findings cannot be shared at this time as the data also forms part of an ongoing study.
